# Physiology

**Published:** 1861-07

**Authors:** S. Weir Mitchell

**Affiliations:** Lecturer on Physiology in the Phila. Med. Association


### PHYSIOLOGY.

BY S. WEIR MITCHELL, M.D.,
LECTURER ON PHYSIOLOGY IN THE PHILA. MED. ASSOCIATION.

### I.—*Alcohol and Anæsthetics.* By LALLEMAND and PERRIN, Paris.

The method which we have followed in studying the physiological effect of alcohol and the anæsthetic agents (chloroform, sulphuric ether, amylene) permits an exact comparison of these bodies with each other, in respect to the successive phases of their action upon the economy.

They exert two kinds of effects : a local action, which is connected with their physico-chemical properties, and a general or physiological action, which does not manifest itself until after their absorption.

Alcohol, which is miscible with water in any proportion, is absorbed by the stomach; if diluted and injected into the veins, it circulates with the blood. Anæsthetics are absorbed only in the gaseous form, and principally by the pulmonary mucous membrane. Sulphuric ether, however, which is more soluble than its two allied substances, is absorbed in the liquid state, at least in part, by the gastric mucous membrane.

Alcohol and the anæsthetics exert an action upon the cerebro-spinal nervous system, which is altogether characteristic.

They produce, in the first place, an excitement, more or less marked, according to their several characteristics. The length of the period of excitement seems to be affected by the solubility and volatility of each of these agents. The duration is longer for alcohol than for either chloroform or amylene, ether occupying a middle place.

Alcohol and anæsthetics, by their gradual effect, at first suspend and finally abolish the sensibility and motor endowments of the nervous system, thus influencing the other functions, and finally causing death.

Alcohol and anæsthetics are neither changed nor destroyed by the body.

They remain for a shorter or longer time in the blood and tissues; the length of their stay being in direct ratio to their solubility, and in inverse ratio to their volatility.

Alcohol and anæsthetics accumulate in the nervous centres by virtue of a special affinity for the tissues of these parts.

The analytic method which we have employed enables us to detect the presence of the smallest quantity of these different agents.

Alcohol can also be distilled in considerable quantities from the organic liquids and solids, and its presence in them thus proven.

The following table indicates the proportion of alcohol and anæsthetics which enter into the composition of the different parts of the body when by ingestion, injection, or inhalation these agents have been received into the system. In each case, the amount found to exist in the blood has been taken as the unit.

| Organic Substances Analyzed. | Alcohol. | | Chloroform. | Sulphuric Ether. | Amylene. |
|---|---|---|---|---|---|
| | Injected into the stomach. | Injected into the veins. | | | |
| Blood | 1 | 1 | 1 | 1 | 1 |
| Brain | 1 34 | 3 | 3·92 | 3·25 | 2·06 |
| Liver | 1·48 | 1·75 | 2·08 | 2·25 | 1 |
| Muscular and cellular tissue | Traces of alcohol. | 1·75 | 0·16 | 0·25 | Traces of amylene. |

Alcohol and anæsthetics are eliminated unaltered in character or amount.

The channel of elimination is governed, to some extent, by the solubility and volatility of these agents. Thus alcohol, a very soluble fluid, passes out in part by the lungs and skin, but chiefly by the kidneys. Ether, less soluble, is eliminated in small amount by the kidneys, but principally by the lungs and skin. Chloroform and amylene, almost insoluble, are thrown out by the lungs and skin alone.

Alcohol, chloroform, sulphuric ether, and amylene may be regarded as a natural family of medicaments nearly allied by their characters and modes of action. They act primitively and directly on the cerebro-spinal nervous system, and are the true anæsthetics.

The carburetted gases, carbonic acid, and carbonic oxide are not anæsthetic agents. They first affect the blood, and through its alteration alone induce, secondarily, phenomena of insensibility. These two latter bodies, therefore, are only false, or indirect anæsthetics.

II.—*Experiments on some of the various Circumstances influencing Cutaneous Absorption.* By A. Waller.

Waller's researches on cutaneous absorption lead to results which appear to us important with regard to practical medicine. The subjects of the experiments were guinea-pigs and albino rats. When the leg of a half-grown guinea-pig was immersed into a mixture of equal parts of chloroform and tincture of aconite, the part was, after fifteen minutes, insensible, and the symptoms of poisoning by aconite soon followed: viz., nausea, efforts at vomiting, coldness of surface, weak circulation, laborious respiration, slight convulsive symptoms, and death. The immersion in simple tincture of aconite without chloroform did not cause any of the symptoms of poisoning. If, however, the sciatic nerve had been previously divided, then the immersion of the leg in the simple tincture of aconite was sufficient to poison the animal, evidently through the distention of the capillaries and the more rapid flow of the blood caused by the division of the nerve. A ligature placed round the limb, before the first symptoms of poisoning had appeared, prevented the toxic influence on the system, but it rarely did so after the appearance of the earliest symptoms. In albino rats, the immersion of the limb in a solution of atropa in chloroform caused dilatation of the pupils after two minutes and a half in young animals, after five minutes or later in old animals. If turpentine was substituted for chloroform, the dilatation of the pupils did not occur while the leg remained immersed in the fluid, but appeared immediately after the removal of the limb. The substitution of alcohol for chloroform as a solvent causes great retardation of absorption. An immersion of twenty to thirty minutes produced only very slight effects. Still more slow is the absorption when the atropa is dissolved in water with a slight addition of acetic acid, or when the watery extract of belladonna is rubbed over the leg. The immersion of the foot of a young rat in a solution of morphia in chloroform caused, after about five minutes, somnolency and a great dilatation of the pupils; which latter phenomenon certainly must appear very remarkable when we consider the effects of morphia, given in the usual way, on the pupil. Three minutes' immersion in a solution of strychnia in chloroform produced dilatation of the pupil, and after five minutes the well-known symptoms of strychnia poisoning manifested themselves. No effect was obtained when a solution of strychnia in alcohol was employed. The experiments clearly show how important is the choice of menstruum in the endermic application of medicinal substances.—(*Proc. of the Roy. Soc.*, vol. x. p. 122; from *The British and Foreign Medico-Chirurgical Review*, Jan., 1861.)

